# Development of Novel Microsatellite Markers for the BBCC *Oryza* Genome (*Poaceae*) Using High-Throughput Sequencing Technology

**DOI:** 10.1371/journal.pone.0091826

**Published:** 2014-03-14

**Authors:** Caihong Wang, Xiaojiao Liu, Suotang Peng, Qun Xu, Xiaoping Yuan, Yue Feng, Hanyong Yu, Yiping Wang, Xinghua Wei

**Affiliations:** 1 State Key Laboratory of Rice Biology, China National Rice Research Institute, Hangzhou, China; 2 College of Agricultural Sciences, Shanxi Agricultural University, Taigu, China; Georgia Institute of Technology, United States of America

## Abstract

Wild species of *Oryza* are extremely valuable sources of genetic material that can be used to broaden the genetic background of cultivated rice, and to increase its resistance to abiotic and biotic stresses. Until recently, there was no sequence information for the BBCC *Oryza* genome; therefore, no special markers had been developed for this genome type. The lack of suitable markers made it difficult to search for valuable genes in the BBCC genome. The aim of this study was to develop microsatellite markers for the BBCC genome. We obtained 13,991 SSR-containing sequences and designed 14,508 primer pairs. The most abundant was hexanuclelotide (31.39%), followed by trinucleotide (27.67%) and dinucleotide (19.04%). 600 markers were selected for validation in 23 accessions of *Oryza* species with the BBCC genome. A set of 495 markers produced clear amplified fragments of the expected sizes. The average number of alleles per locus (*Na*) was 2.5, ranging from 1 to 9. The genetic diversity per locus (*He*) ranged from 0 to 0.844 with a mean of 0.333. The mean polymorphism information content (PIC) was 0.290, and ranged from 0 to 0.825. Of the 495 markers, 12 were only found in the BB genome, 173 were unique to the CC genome, and 198 were also present in the AA genome. These microsatellite markers could be used to evaluate the phylogenetic relationships among different *Oryza* genomes, and to construct a genetic linkage map for locating and identifying valuable genes in the BBCC genome, and would also for marker-assisted breeding programs that included accessions with the AA genome, especially *Oryza sativa*.

## Introduction

The *Oryza* genus comprises more than 22 species with 10 recognized genomic types, six of which are diploid genome sets (2n =  24, AA, BB, CC, EE, FF, and GG) and four of which are tetraploid (2n =  4x =  48, BBCC, CCDD, HHJJ, and HHKK) [1]. According to their genome constitution, species in this genus can be classified into four main complexes [2]: *Oryza ridleyi* complex (including the HHJJ genome); *Oryza granulate* complex (including the GG genome); *Oryza officinalis* complex (including the BB, CC, BBCC, CCDD, and EE genomes); and *Oryza sativa* complex (including the AA genome). There are two cultivated *Oryza* species, referring to *Oryza sativa* and *Oryza glanerrima*. Asian cultivated rice (*Oryza sativa*) is one of the most important food crops in the world, and serves as a primary food source for more than half of the world's population [3]. In the field, cultivated rice plants are continuously damaged by various biotic and abiotic factors. The planting of modern varieties with resistance and/or tolerance genes is one of the best strategies to control pests in rice production. Some populations of wild species of *Oryza* have been identified as extremely valuable resources that can be used to broaden the genetic background of cultivated rice to increase its resistance to adverse factors.

The BBCC *Oryza* genome (2n =  4x =  48) is characteristic of allotetraploid wild species with two homologous genomes, B and C. Three species have this genome type: *Oryza malampuzhaensis*, which is found in India; *Oryza minuta*, which is endemic to Philippines and Papua New Guinea; and *Oryza punctata* (tetraploid, 2n =  48), which is widely distributed in Africa. The BBCC genome is related to the BB and CC genomes [1]. Only *Oryza punctata* (2n = 24) has the BB genome [4], [5], while *Oryza officinalis*, *Oryza rhizomatis* and *Oryza eichingeri* have the CC genome. These species are regarded as donors of genes that promote resistance to rice blast, bacterial leaf blight, brown planthopper, and white backed planthopper [6], [7].

However, the transfer of valuable genes from these wild species to *Oryza sativa* via crossing has been proved to be extremely difficult because of low seed set, hybrid sterility, and the lack of chromosome recombination [8]. There is no doubt that appropriate gene identification technologies will promote the use of genetic material from these wild species. The traditional method to identify the genomes of *Oryza* was to observe chromosome pairing behavior at meiotic metaphase-I in interspecific hybrids [9], [10]. However, this process was affected by genetic and environmental factors [11], [12]. Subsequently, genomic *in situ* hybridization (GISH) was used to identify genomes [13], followed by multicolor genomic *in situ* hybridization (McGISH), an improved method that used two different genomic probes simultaneously [14]. Both GISH and McGISH were complex methods with highly technical requirements. More recently, DNA molecular techniques, especially simple sequence repeat (SSR) markers, have been proved to be simple and highly effective methods for genetic analysis. A large number of SSR markers have been developed for *Oryza sativa* [15], [16]. While some of the SSRs developed for *Oryza sativa* could be amplified from other AA genomes in the *Oryza* genus, they were not suitable for cross-amplifications from *Oryza* species with different genome types [17], as preceding cross-amplifications by *Miscanthus sinensis* (*Poaceae*) and its relative [18] and *Narcissus papyraceus* (*Amarillydaceae*) and its relatives [19]. Since there had being no sequence information available for the BBCC genome, no special markers have been developed for it. This made it difficult to explore the BBCC genome to find valuable genes, and to study the phylogenetic relationships among diverse members of the *Oryza* genus.

Hence, the goal of this study was to develop the first set of microsatellite markers for the BBCC *Oryza* genome using next generation sequencing (NGS) technology. These microsatellite markers could be used to evaluate the phylogenetic relationships among different *Oryza* genomes, and to construct a genetic linkage map for locating and identifying valuable genes in the BBCC genome, and would also for marker-assisted breeding programs that include accessions with the AA genome, especially *Oryza sativa*.

## Materials and Methods

### Plant materials and DNA extraction

We chose seven *Oryza* species including 48 accessions (Table S1) in this study, referring to different ploidy levels, genomic constitutions, and genome origins. 38 accessions were obtained from the Germplasm Resource Center of the International Rice Research Institute (Los Banos, Philippines), including 23 accessions with the BBCC genome, 1 with the BB genome, and 14 with the CC genome. The other 10 accessions of *Oryza sativa* were obtained from the National Mid-term Genebank for Rice (Hangzhou, China).

Total genomic DNA was extracted from fresh leaves using the DNeasy Plant Mini Kit (Qiagen, Valencia, CA, USA).

### Microsatellite loci search and SSR primer development

Genome libraries were constructed from the accession W303 (*Oryza minuta*) based on shotgun method, and then sequenced using the Illumina Hi Seq 2000 sequencer (Illumina Inc., San Diego, CA, USA). The genome of W303 (European Bioinformatics Institute; Accession number: PRJEB5091) was assembled using Phusion2 [20] and Phrap [21]. The N50 length of the entire assembly was calculated for the initial contigs with small contigs <1000 bp excluded.

The SSRs were identified by the software MISA (Microsatellite identification tool, http://pgrc.ipk-gatersleben.de/misa/). The primers for each unique SSR were designed using the Primer 3.0 (http://sourceforge.net/projects/primer3/). The primer design parameters were as follows: length from 18 bp to 23 bp with 21 bp as the optimum; annealing temperature between 55°C and 63°C with 60°C as the optimum; GC content from 40% to 60% with 50% as the optimum; and PCR product size between 80 bp and 250 bp.

### SSR genotyping

The PCR amplifications were carried out with a 2720 thermal cycler (Applied Biosystems, Foster City, CA, USA) in 10 μL reaction mixtures. Each reaction contained 1.0 μL 10× buffer, 1.0 μL 2 mmol/L dNTPs, 1.0 μL 25 mmol/L MgCl_2_, 0.6 μL each of forward and reverse primer (10 μmol/L), 0.1 μL 5 U/μL Taq polymerase, and 20 ng template DNA. The PCR cycling profile was as follows: 94°C, 2 min; 35 cycles of 94°C, 30 s, 60°C with a increase/decrease of 1°C, 30 s, and 72°C, 1 min; and 72°C, 8 min. The amplification products were analyzed by an Applied Biosystems 3130xl DNA analyzer (Applied Biosystems), and the data were processed using GeneScan and GeneMapper software (Applied Biosystems).

### Statistical analysis

The average number of alleles per locus (*Na*), the genetic diversity per locus (*He*), and the polymorphic information content (PIC) were calculated with the Powermarker Software [22]. All 48 accessions were clustered using the Neighbor-Joining (NJ) tree implemented in the TreeView program [23] according to the *Nei*'s unbiased genetic distance [24] with 100 bootstrap replications, using the *Oryza sativa* as an out-group.

## Results

### Data from sequencing and microsatellite loci detected

As shown in Table 1, a total length of the assemble sequences >1000 bp was 480,470,380 bp (n = 225,883) (http://www.ricedata.cn/down/W303_fasta.rar). The average length of the read sequences was 2,128 bp, with a maximum length of 41,615 bp and no sequences shorter than 1,000 bp.

**Table 1:** The statistics about the sequence assembly.

Category	Length (bp)	Number
sum	480,470,380	225,833
ave	2,128	
largest	41,615	
N50	2,329	65,627
N90	1,203	182,019

In total, 16,197 SSR loci were identified with discrete repeats accounting for 97% and compound repeats (C* type and C type) accounting for only 3%. We obtained 13,991 SSR-containing sequences, and 1,814 sequences contained more than one SSR. There were 503 SSRs present in compound formation (Table 2). Finally, 14,508 primer pairs were designed.

**Table 2:** Occurrence of the sequence analysis and microsatellites in the genome survey.

Category	Numbers
Total number of sequences examined	225,833
Total size of examined sequences (bp)	480,470,380
Total number of identified SSRs	16,197
Number of SSR containing sequences	13,991
Number of sequences containing more than 1 SSR	1,814
Number of SSRs present in compound formation	503

### Distribution of identified microsatellite motifs and classified repeat types

**Figure 1:**
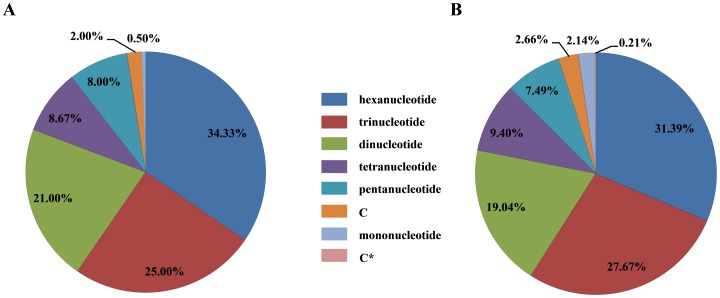
Frequencies of different classes of nucleotide repeats. (A) 14508 primer pairs; (B) 600 selected primer pairs.

We set the following minimum length criteria in MISA to extract repeated units (unit size/minimum number of repeats): (1/18), (2/9), (3/6), (4/5), (5/4), and (6/3). The SSR motif of hexanucleotide repeats (5,090, 31.4%) was the most abundant class, followed by trinucleotide (4,529, 28.0%), dinucleotide (3,131, 19.3%), tetranucleotide (1,603, 9.9%), pentanucleotide (1,182, 7.3%) and mononucleotide repeats (662, 4.1%) (Figure 1a); the SSR motif detected at the highest frequency in each class was ATCTTT, CGC, CT, TATG, AATCT, and G, respectively. The most abundant SSR repeat type in each class was AAAAAG/CTTTTT (4.0%), AGG/CCT and CCG/CGG (16.3%), AG/CT (74.6%), ACAT/ATGT (13.7%), AGAGG/CCTCT (9.7%) and C/G (64.7%), respectively.

### Characterization of microsatellite markers for the BBCC genome

We designed 14,508 primer pairs, and selected a set of 600 SSR markers based on proportional distribution (Figure 1). We tested the ability of the 600 primer sets to amplify SSRs from 23 accessions with the BBCC genome. Of the 600 primer pairs, 50 did not produce amplicons, probably because of mutations at the SSR locus. 55 did not amplify fragments of the expected size, probably because of In/Del mutations at the SSR locus. Of the remaining 495 microsatellite markers (Table S2, http://www.ricedata.cn/down/SSR_data.xlsx), 156 were monomorphic, and 339 were polymorphic. There were 223 single copy and 272 multi-copy markers. The mean *Na* value was 2.5 with a range from 1 to 9. The *He* value varied from 0 to 0.844 with a mean of 0.333. The mean PIC was 0.290, and ranged from 0 to 0.825. Among these markers, 46 were unique to *Oryza minuta*, five were unique to *Oryza punctata*, and none were specific to *Oryza malampuzhaensis*. The genetic diversity of *Oryza minuta* was lower than that of *Oryza punctata* (Table 3; *Na* = 1.4 vs. 1.4; *He* = 0.093 vs. 0.125; *PIC* =  0.081 vs. 0.102).

**Table 3:** Details of 46 and 5 microsatellites specific to *Oryza minuta* and *Oryza punctata*, respectively.

Locus	Repeat motif	Forward primer (5'-3')	Reverse primer (5'-3')	Tm(°C)	Expected product size (bp)	*Oryza minuta* genetic characterization				*Oryza punctata* genetic haracterization			
						Especially	*Na*	*He*	*PIC*	Especially	*Na*	*He*	*PIC*
CN001	(CAGCT)4	TACAAGTGGGCTTAGGGTGG	GTCGAGCCAGTTCGTTATCC	60	100	√	2	0.298	0.253				
CN019	(CTTTTC)4	ATCCACATGGCAAACTACCC	ACATCTTTTGCCACACATCG	60	115	√	1	0.000	0.000				
CN026	(AATT)5	AATGTGGATTAGGCACGAGG	ACGGGCATACTAATCAACGC	60	120					√	2	0.444	0.346
CN028	(CTTTAT)3	CGCACGTTAATATCACCTCG	GAAGACAATTCTGGTCGATGG	60	120	√	1	0.000	0.000				
CN032	(CTCCGT)3	GATCGATCCTTCTGGAACCC	CAGTCGGAGGAGAAAAGTGC	60	122					√	2	0.180	0.164
CN036	(ATCTAT)3	ATAGATCCCACGTGTCAGCC	GTCTTGGACTCGGATTTTGC	60	125	√	1	0.000	0.000				
CN040	(AAAC)5	GCAGTCATCGAGTCCCTAGC	TGCTTACTCATCATCCTGCG	60	128	√	1	0.000	0.000				
CN049	(TC)14	TACACGCCTTTTGTTCTTCG	CAACGATGATTATGATGCGG	60	133					√	1	0.000	0.000
CN066	(AAGATA)3	ACCTGCATCCTACACTTGCC	TATTGTCACCTCGTTTTGCG	60	146	√	3	0.292	0.272				
CN070	(GAATCG)3	TAAGGATGAAAACCGCTTGG	CCGTATTTGCTCAGTTTTCG	58	148	√	1	0.000	0.000				
CN079	(AGAGGG)3	AATCTGTCAATGGGCAGTCC	CGCAACTCACATAGAAACGG	59	153	√	3	0.512	0.444				
CN081	(CTG)6	AATGCACAACAAGTCTCCCC	ATCTGGAAGGAGCAATGACG	60	154					√	1	0.000	0.000
CN082	(AAAATG)3	ATTTTGTTCCGATGGTCTCG	GTTAGGGATGAAAACGGTCG	59	154	√	1	0.000	0.000				
CN093	(CAAC)5	TTGGTGATCGAGCACATAGC	AGATTGATTCACATGCGTGG	60	159	√	1	0.000	0.000				
CN099	(AAAGG)4	TCTGTGGATCACAAGCAAGC	ATAAAAAGGGAAGGCATCCG	60	161	√	1	0.000	0.000				
CN102	(ATATAC)3	TGGAGGGTTATAATCAGCGG	AAGACATTGGAGCTTGACCG	60	161	√	2	0.444	0.346				
CN108	(TTTAT)5	GGGGAGAAATACCGGTAAGC	ACCTCACATCTCAACCCTGC	60	165					√	1	0.000	0.000
CN124	(TTGTG)4	ATTCAGATTCACCTCCGACG	ACCCACGAAAAGGTGTATCG	60	169	√	1	0.000	0.000				
CN150	(CGC)8	TAATCCGAGGACCAAAGTGC	CTGAGCGTAGGATGAGGAGG	60	178	√	4	0.597	0.552				
CN156	(AGG)6	ACGTCGACCTCTTCACAAGC	CTTCCTCCACAGCTCACTCC	60	179	√	2	0.298	0.253				
CN171	(ATGAAG)3	GGAGCACATGGAAGAGAAGC	AATGGATTTCTCGTTTTGCG	60	184	√	1	0.000	0.000				
CN174	(T)18	TACCAGCTCCTTCTGATGGC	ACTTGTTAATCCAGTGGCGG	60	185	√	1	0.000	0.000				
CN181	(CAACGG)3	TCTGACGATGCAATCAAAGC	TTGTCGTAAGCAGCAACTCG	60	185	√	1	0.000	0.000				
CN189	(CCTCGT)3	ATCGATGCACACTCAACTGC	ACAATGATGGAGAGGAACGC	60	188	√	2	0.153	0.141				
CN206	(CCAAAT)3	TGCCATATTTGAGCTGATGC	CAAGAGATGGAGGAGCAAGG	60	194	√	1	0.000	0.000				
CN207	(CGGTCA)3	GGCTTAAAACCAAACCCTCC	TTGTGTAGTGAGGCGAGTGC	60	194	√	1	0.000	0.000				
CN208	(TC)9	AACCCTAGTTTTCCCATCCG	AGGAGCCGATCTAGAGGTCC	60	195	√	3	0.542	0.460				
CN210	(ACAT)5acacacat(ATAC) 7atatatatgtgtgtg(TATGTA)3	ATCGGTATCATATGCAGCGG	TTTGCTACATCCAACATGTGC	60	198	√	1	0.000	0.000				
CN212	(GCGAG)4	TATGTCTCGTCACAGCTGGC	GAGACGGGTAGGTAGGGAGG	60	198	√	1	0.000	0.000				
CN217	(CCAGCG)3	GGCTCCTGAAAACAATCTGC	TTCCAATCTCTCCCATCTCG	60	199	√	1	0.000	0.000				
CN253	(TCTTC)4	GGACGAAAAACCTAATCCCC	TAACACTGATCCGCACAACG	61	208	√	2	0.165	0.152				
CN275	(CAACGG)3	CTCTTATGCCAAATCCGACG	GCATTTTGGTATTTCCACCG	60	211	√	2	0.153	0.141				
CN281	(GCGA)5	CATGATTGAACTGGTGACCG	GCGTGGGTAGAGAGAGATGG	60	213	√	1	0.000	0.000				
CN283	(CT)9	GATGAGGGTGACAGAGAGGC	AGTGTATCTTGCTCCACCGC	60	214	√	1	0.000	0.000				
CN287	(CTTTAT)3	CGCACGTTAATATCACCTCG	TTAAGAAGGCAAATCGGAGC	59	214	√	1	0.000	0.000				
CN300	(CAATGG)3	GAGACAGCCAACTCCTACGC	TCGGCTACATTGTGTGAAGC	60	218	√	1	0.000	0.000				
CN310	(CCG)6	TGGGAATGAGAAGGAAGACG	TGTCCGCTACTACTGATGCG	60	221	√	1	0.000	0.000				
CN330	(AGTGCT)3	CCTCCTGCTTCACAAACTCC	ACGATATGCTCCCATGTTCC	60	225	√	1	0.000	0.000				
CN348	(CGCCCG)3	ACCTTCCTCCTCAACTTCCC	CTTGAAAATTCGGGTTAGCG	60	229	√	1	0.000	0.000				
CN356	(CTTCAG)3	CTCAACAGTTCAAATGGATTGC	TTTGTGCTGTGAAAGCAAGG	60	230	√	1	0.000	0.000				
CN379	(ATCC)5	ATCGCTTCTCTCCCTTAGCC	AAATGCTCAGTGGGTTTTGG	60	235	√	1	0.000	0.000				
CN387	(CTC)7	TGTCGTTGTCACCTTCTTCG	GCGAGAATAAGCTGTTTGGC	60	237	√	1	0.000	0.000				
CN391	(AGA)9	AGTGGGCTACATGAGATGGC	GCATCCTGTTCTTGACCACC	61	238	√	2	0.153	0.141				
CN418	(CGGAAA)4	TCTGCTGTGGTAAAAACCCC	TTTCGAAAAGTTTCCAACCG	60	241	√	2	0.153	0.141				
CN434	(CTAGCT)3	GGAAGCTCATGACAACCTCC	CTCTTGTTGAGCTTGCCTCC	60	245	√	1	0.000	0.000				
CN453	(GTC)6gctcccttgctcctcgccat gcttcgggtgaagctggcgagg(GTG)6	CTGATCACCATGTACCACGC	GAACCTGTCACCGATCATGG	61	251	√	3	0.500	0.449				
CN467	(ATCTAA)3(TCTAAA)4	TGCAAAAGAAAAGCTAATCGG	GGAAAAGAAACGTGCAAAGC	60	267	√	1	0.000	0.000				
CN476	(GA)11	AAAAATTGTCTGCCGTCTGG	AACCCTAGAATTCCCATCCG	60	271	√	1	0.000	0.000				
CN478	(AATAGG)3	ACCAATCTTGGGAAGACACG	TCCCTTCTCATATCGCATCC	60	271	√	1	0.000	0.000				
CN486	(AAAAGT)3	AAACCTTGGAAAAGGCTTGG	CTTGAGGAGTCCACTCTCCG	60	276	√	1	0.000	0.000				
CN490	(TG)9	GAATGGCTGTGTCAATGTGG	ATGACTCATCTCAATCGGGC	60	279	√	1	0.000	0.000				
Mean							1.4	0.093	0.081		1.4	0.125	0.102

### Cross-amplification from other related genomes

Next, we evaluated the suitability of these 495 markers for use in other closely related species. Of the 495 markers, only 12 (2.4%) were specific to the BB genome, 173 (34.9%) were specific to the CC genome, and 299 (60.4%) were common to the BB, CC, and BBCC genomes. Eleven markers (2.2%) were neither in the BB nor the CC genome. Most interestingly, 198 markers (40.0%) were also present in the AA genome.

**Figure 2:**
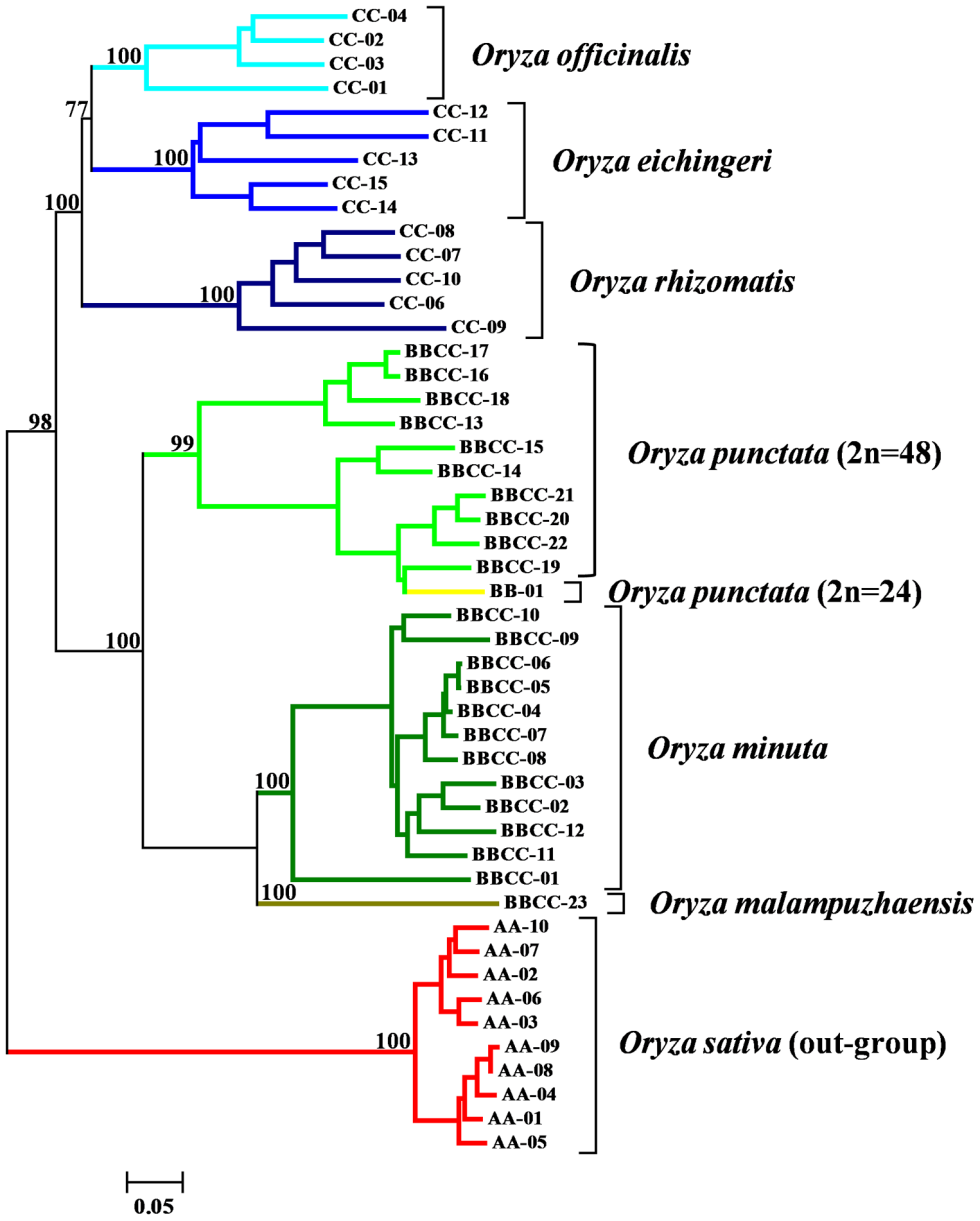
Neighbor-Joining tree of 48 accessions based on *Nei*'s unbiased genetic distance from 495 SSR markers. Bootstrap values (out of 100) are indicated at the branch points.

The phylogenetic tree (Figure 2) grouped the 48 accessions into two significant, distinct clusters. Cluster I consisted of the BB, CC, and BBCC genome species; and cluster II consisted of the AA genome species. Cluster I was further divided into two groups, one consisting of species with the BBCC and BB genomes, and the other consisting of species with the CC genome. Within the BBCC genome, *Oryza minuta* and *Oryza punctata* formed different subgroups. *Oryza malampuzhaensis* was more closely related to *Oryza minuta* than to *Oryza punctata*. Among the species with the CC genome, *Oryza eichingeri* was more closely related to *Oryza officinalis* than to *Oryza rhizomatis*. In cluster II, *Oryza sativa indica* and *Oryza sativa japonica* were clearly divided into two groups. The groups in the NJ tree were consistent with the intrinsic relationships among *Oryza* species [17], and further confirmed the usefulness of the new developmental microsatellite markers in genetic analyses.

## Discussion

We developed the first set of microsatellite markers for the BBCC *Oryza* genome. The SSRs were located in both coding and non-coding regions, and therefore, they would be useful for genetic and evolutionary analyses, high-throughput mapping, and marker-assisted plant improvement strategies. In this study, 82.5% of selected markers produced clear amplified fragments of the expected sizes. This was similar to the success rate of 60–90% amplification reported elsewhere [25]. Among these markers, 12 were specific to the BB genome and 173 were unique to the CC genome. Thus, these unique microsatellite markers could be developed as probes to identify different species and various genomes. We evaluated the transferability rates of the markers in different *Oryza* species. The transferability rate between *Oryza minuta* and *Oryza punctata* was 89.7%. This was higher than that for *Oryza* species with the BB, CC, and BBCC genomes (60.4%), and that between AA and BBCC genomes (40.0%). These high transferability rates suggested that different species or genomes within the *Oryza* genus were closely related.

Our results showed that hexanucleotide repeat motif (31.4%) was the most abundant repeat type, followed by trinucleotide (28.0%) and dinucleotide (19.3%). These findings differed from those of previous studies in which dinucleotide or trinucleotide repeats were reported to be the most abundant motifs in genomes of cultivated rice [16], [26], and pentanucleotide repeats (30.5%) were the most abundant type in *Gossypium raimondii* [17]. The nature of the microsatellites obtained was related not only to the thresholds used to define the microsatellites, but also to genome organization, since heterogeneity could lead to differences in microsatellite size [27]. The most common hexanucleotide motif was AAAAAG/CTTTTT (4.0%), which made up a much lower proportion than that of the most common motif in *faba* bean, ACACGC/CGTGTG (49.5%) [28]. The main trinucleotide repeats were AGG/CTT and CCG/CGG, representing 16.3% of all of the trinucleotide repeats analyzed. The most common trinucleotide repeats were AGG/CTT in *Amorphophallus* [25], and CGG/GCC in cultivated rice [16], [26]. These results provided further evidence that the CCG/CGG motif was very common in monocots [29]. This reflected the strong conservation of synteny among genomes of diverse monocots, and could result from a high GC content and codon bias [30], [31].

In previous studies, mitochondrial restriction fragment length polymorphisms (RFLPs) [32] and inter simple sequence repeat (ISSR) [33] markers had been used to study genetic relationships among members of the *Oryza* genus. However, these analyses could only distinguish the AA genome from other types, and could not separate other related genomes, such as the BB, CC, and BBCC genomes. In contrast, the SSR markers developed from the BBCC genome were able to differentiate the AA, BB, CC, and BBCC genomes, and also distinguished the BB and CC genomes from the BBCC genome, even identified various species within the AA, CC, and BBCC genomes. Thus, the relationships predicted from analyses using these markers were consistent with the established evolutionary relationships among members of the *Oryza* genus [17]. Despite this, a new marker, SNP (Single Nucleotide Polymorphism), is now on the scene and has gained increasing popularity. In terms of genetic information provided, as simple bi-allelic co-dominant markers, they can be considered as a step backwards when compared to the highly informative multi-allelic microsatellites [34].

The NJ tree further revealed that the BB genome species were more closely related to species with the BBCC genome than to those with the CC genome, demonstrating that the BB genome was the maternal parent of the BBCC genome [35], [36] and CC species evolved later [37]. *Oryza malampuzhaensis* and *Oryza officinalis*, both of which had the BBCC genome, shared similar morphologies; in fact, *Oryza malampuzhaensis* was considered to be a subspecies of *Oryza officinalis* [38]. There were clear differences in the panicle and spikelet between these two species [14]. Our results showed that *Oryza malampuzhaensis* was more closely related to *Oryza minuta* than to *Oryza officinalis*, consistent with the fact that *Oryza malampuzhaensis* was an allotetraploid with the BBCC genome [39] while *Oryza officinalis* was a diploid with the CC genome.

## Conclusions

We present the first set of microsatellite markers from the nuclear BBCC *Oryza* genome. Our results showed that the high-throughput approach for sequencing was useful for obtaining many high quality SSR markers. These markers can be used to study the origins and evolutionary relationships among members of the *Oryza* genus, and could also be used to construct physical maps and for map-based gene cloning from the BBCC genome to identify valuable genes. Furthermore, they could be used for marker-assisted trait selection in cultivated rice breeding programs. By using the pre-existing sequence information, the further analysis will focus on the SNPs development which is known as a new marker.

## Supporting Information

Table S1 ***Oryza*** **species and accessions used in this study.**
(XLS)

Table S2 **Characteristics of 495 microsatellite markers producing clear amplified fragments of the expected sizes.**
(XLS)
